# Achieving Effective Multimodal Imaging with Rare-Earth Ion-Doped CaF_2_ Nanoparticles

**DOI:** 10.3390/pharmaceutics14040840

**Published:** 2022-04-11

**Authors:** Zhenfeng Yu, Yuanyuan He, Timo Schomann, Kefan Wu, Yang Hao, Ernst Suidgeest, Hong Zhang, Christina Eich, Luis J. Cruz

**Affiliations:** 1 Translational Nanobiomaterials and Imaging Group, Department of Radiology, Leiden University Medical Center, 2333 ZA Leiden, The Netherlands; z.yu@lumc.nl (Z.Y.); y.he@lumc.nl (Y.H.); t.schomann@lumc.nl (T.S.); y.hao@lumc.nl (Y.H.); 2 Percuros B.V., Zernikedreef 8, 2333 CL Leiden, The Netherlands; 3 Van’t Hoff Institute for Molecular Sciences, University of Amsterdam, Science Park 904, 1098 XH Amsterdam, The Netherlands; k.wu@uva.nl (K.W.); h.zhang@uva.nl (H.Z.); 4 C.J. Gorter Center for High Field MRI, Department of Radiology, Leiden University Medical Center, 2333 ZA Leiden, The Netherlands; e.suidgeest@lumc.nl

**Keywords:** rare-earth ions, multimodal imaging, NIR-II fluorescence, photoacoustic, magnetic resonance

## Abstract

Nowadays, cancer poses a significant hazard to humans. Limitations in early diagnosis techniques not only result in a waste of healthcare resources but can even lead to delays in diagnosis and treatment, consequently reducing cure rates. Therefore, it is crucial to develop an imaging probe that can provide diagnostic information precisely and rapidly. Here, we used a simple hydrothermal method to design a multimodal imaging probe based on the excellent properties of rare-earth ions. Calcium fluoride co-doped with ytterbium, gadolinium, and neodymium (CaF_2_:Y,Gd,Nd) nanoparticles (NPs) is highly crystalline, homogeneous in morphology, and displays a high biosafety profile. In addition, in vitro and ex vivo experiments explored the multimodal imaging capability of CaF_2_:Y,Gd,Nd and demonstrated the efficient performance of CaF_2_:Y,Gd,Nd during NIR-II fluorescence/photoacoustic/magnetic resonance imaging. Collectively, our novel diagnosis nanoparticle will generate new ideas for the development of multifunctional nanoplatforms for disease diagnosis and treatment.

## 1. Introduction

To date, countries around the world are facing major challenges posed by cancer. Cancer is a highly recurrent and lethal disease with low cure rates, a continuous increase in patients, inadequate performance of medical equipment, and the serious shortage of medical staff, which have led to the gradual overdraft of the medical system. Consequently, an emerging hot topic is the efficient diagnosis of the condition, as well as quick and rational allocation of medical resources and the reduction of the medical burden. In recent decades, the main diagnosis and treatment methods for the early disease stages included magnetic resonance imaging (MRI) [1,2], X-ray computed tomography (CT) [3,4], positron emission tomography (PET) [5,6,7], single-photon emission computed tomography (SPECT) [8,9], magnetic particle imaging (MPI) [10,11], and optical fluorescent light imaging (FLI) [12,13]. It is worth noting that photoacoustic imaging (PAI) is expected to become a powerful tool for clinical detection due to its super-spatial resolution, high penetration, and non-invasive imaging technology [14,15]. The application of these detection tools can provide relevant information about patients’ diseases and help doctors formulate treatment plans. However, in addition to the advantages of diagnostic tools, each imaging modality has its own disadvantages that cannot be ignored, such as low temporal and/or spatial resolution, low sensitivity, long response time, etc. [16,17,18]. This determines that each method can only provide information on a specific aspect, and usually multiple tests are required to combine all the diagnostic information to obtain a relatively comprehensive diagnosis. This is not only time consuming, but also causes a serious waste of medical resources. Thus, the development of an imaging probe that integrates multiple detection functionalities can improve the efficiency of medical diagnosis and alleviate the dilemma faced by the current medical system.

Due to the excellent physical properties of rare-earth elements, i.e., their optical, electrical, and magnetic characteristics, the doping of rare-earth elements with other materials can lead to the formation of new materials with different properties. In this way, rare-earth materials are gradually becoming a focus for research and development of high technology, with application in the treatment and diagnosis of tumors [19]. In addition, rare-earth elements contain unfilled 4f electrons, and the 4f-layer electrons are shielded by the outer layer of 5S^2^, 5P^6^ electrons, which can easily jump between the high energy levels, forming an extremely complex spectrum. This property has laid the foundation for rare earth elements to become superior luminescent materials. Novel trivalent rare-earth ion-doped, inorganic nanomaterials have attracted a great deal of attention in recent years [20,21,22,23,24]. In particular, fluorides with low phonon energy and high chemical stability are considered ideal host materials for lanthanide-doped down-conversion nanomaterials such as SrF_2_ [25,26,27], CaF_2_ [28], NaYF_4_ [29,30], NaLuF_4_ [31,32], and NaGdF_4_ [33,34,35]. Compared to other fluorides, CaF_2_ has better biocompatibility and high optical transparency, and can effectively prevent the leakage of rare-earth ions, reducing the possibility of diseases, such as nephrogenic systemic fibrosis [36,37,38,39]. It has been established that water has a huge absorption peak during the upconversion process under 980 nm excitation, which can easily cause an overheating effect and damage the organism [40]. However, if the excitation wavelength of the nanomaterial is located in the first near-infrared (NIR) window (650–900 nm), it can effectively reduce light damage, and water reaches its lowest local absorption value around 800 nm. Therefore, we chose Nd^3+^ as the main NIR emitter of the new material, which has a strong absorption cross-section at a wavelength of 808 nm [41,42,43,44,45,46]. In order to obtain novel materials with high quantum efficiency, one of the issues we have to address is the quenching effect of the luminescence intensity caused by the dose of Nd^3+^ doping [47,48]. A considerable amount of literature has been published regarding the use of Y^3+^ as a modulating ion for the co-doping of Nd^3+^:CaF_2_ crystals [49,50,51,52]. These studies have confirmed that Y^3+^ as a dopant can effectively destroy the Nd-Nd clusters in the CaF_2_ structure, leading to improved material performance. Recently, Li et al. investigated the combined effect on the spectral properties of Nd^3+^,R_1_^3+^,R_2_^3+^:CaF_2_ (R_1_, R_2_ = Y, La, Gd, Lu) crystals in detail by co-doping several non-optically active regulatory ions into Nd^3+^:CaF_2_ crystals, and demonstrated that Nd^3+^,Y^3+^,Gd^3+^:CaF_2_ crystals have excellent laser properties [53]. Therefore, we chose a combination of Y^3+^, Gd^3+^, and Nd^3+^ to explore the performance of small-sized CaF_2_ nanoparticles (NPs). In addition, the well-known exceptional paramagnetic properties of Gd^3+^ may also help the NPs to achieve MRI with excellent spatial resolution. Therefore, the combination of Y^3+^, Gd^3+^, and Nd^3+^ was chosen to explore the performance of small-sized CaF_2_ NPs.

**Figure 1:**
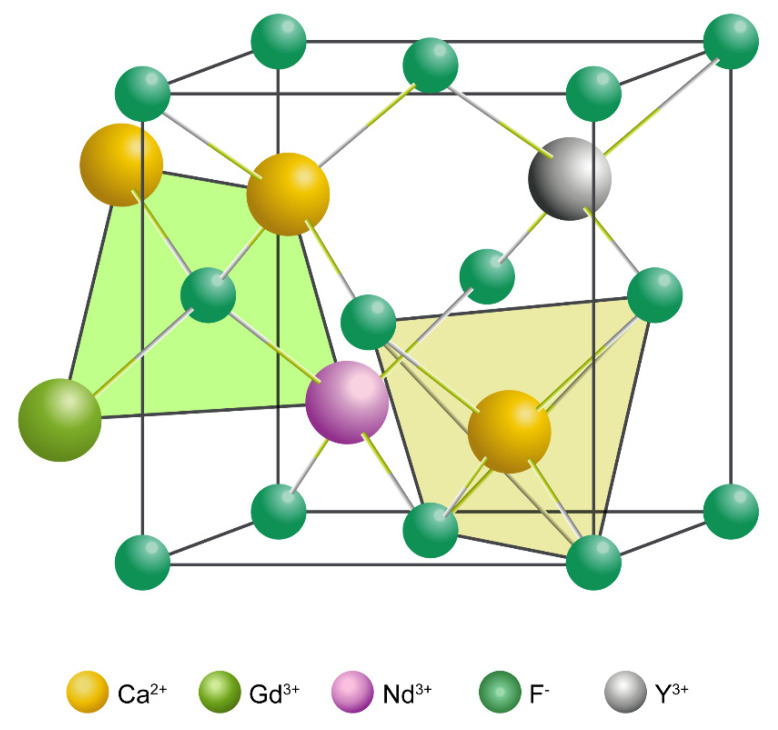
Predicted three-dimensional (3D) structure of CaF_2_:Y,Gd,Nd NPs.

In the present work, we demonstrate a simple synthesis strategy to achieve “three-in-one” imaging of NPs by co-doping rare earth ions (Y^3+^, Gd^3+^, and Nd^3+^) with diverse properties into a CaF_2_ matrix using a hydrothermal method (Figure 1). This convenient and efficient synthesis method not only results in NPs with multimodal imaging properties (NIR-II/PA/MRI), but also avoids material loss and performance degradation observed with other multimodal imaging materials due to complex synthesis processes. In addition, the obtained CaF_2_:Y,Gd,Nd NPs display uniform morphology, excellent biocompatibility, as well as outstanding imaging properties. The doping of Y^3+^ and Gd^3+^ not only breaks the concentration quenching threshold of Nd^3+^, but also enhances the NIR-II luminescence properties and paramagnetic properties of the NPs, thereby enabling NIR-II and MR imaging of CaF_2_:Y,Gd,Nd NPs. Meanwhile, the reaction of the three doping elements endows CaF_2_:Y,Gd,Nd NPs with unique photoacoustic properties. In conclusion, our novel, multimodal-imaging material can simultaneously achieve high temporal and spatial resolution.

## 2. Materials and Methods

### 2.1. Materials

All chemicals were ready for use without additional purification. Calcium chloride dihydrate (CaCl_2_·2H_2_O, 99.5%), yttrium (III) chloride heptahydrate (YCl_3_·6H_2_O, 99.99%), neodymium (III) chloride hexahydrate (NdCl_3_·6H_2_O, 99.9%), gadolinium (III) chloride hexahydrate (GdCl_3_·6H_2_O, 99.9%), ammonium fluoride (NH_4_F, ≥98.0%), potassium citrate tribasic monohydrate (HOC(COOK)(CH_2_COOK)_2_·H_2_O, ≥99.0%), and ethylenediaminetetraacetic acid (EDTA) were purchased from Sigma-Aldrich (St. Louis, MO, USA); 4′,6-diamidino-2-phenylindole (DAPI), Dulbecco’s modified eagle’s medium (DMEM), MHC II-PE, CD86-FITC, and fetal calf serum (FCS, Gibco Laboratories, Brooklyn, NY, USA) were purchased from Thermo Fisher Scientific (Waltham, MA, USA); CellTiter 96 AQueous MTS reagent powder was purchased from Promega (Madison, WI, USA); lipopolysaccharide (LPS) was purchased from PeproTech (Cranbury, NJ, USA); agarose was purchased from Bioline (London, UK); phalloidin-iFluor 488 Reagent was purchased from Abcam (Cambridge, UK); CD40-APC was purchased from Biolegend (San Diego, CA, USA). All experimental water was deionized (DI) water.

### 2.2. Synthesis of the CaF_2_:Y,Gd,Nd NPs

CaF_2_:Y,Gd,Nd NPs were prepared using a hydrothermal method. In a 100 mL beaker, a total of 3.75 mmol of stoichiometric CaCl_2_·2H_2_O, YCl_3_·6H_2_O, GdCl_3_·6H_2_O, and NdCl_3_·6H_2_O (Ca^2+^:Y^3+^:Gd^3+^:Nd^3+^ = 0.68:0.15:0.15:0.02) was dissolved in 7 mL of DI water. A total of 20 mL (6.4882 g) sodium citrate was added dropwise to the above solution and stirred vigorously at 1500 rpm for 20 min (min using a magnetic stirrer (IKA, Staufen, Germany)). Then, NH_4_F (0.3241 g) in aqueous solution was added to the previous solution. The obtained clear solution was placed in a Teflon-lined autoclave (Baoshishan, China) and treated at 180 °C for 10 h (h). The autoclave was then cooled to room temperature and the NPs were collected by centrifugation (2.4× *g*, 20 min), washed 3 times with water and ethanol, and then dried using a freeze dryer (Martin Christ, Osterode, Germany).

### 2.3. Characterization

Powder X-ray diffraction (XRD) patterns were measured using a Panalytical X’pert PRO powder diffractometer (Malvern Panalytical, Malvern, UK) with a CuKα (λ = 1.5405 Å) source operating at 40 kV and 40 mA in steps of 6.0° and a 2θ range of 10° ≤ 2θ ≤ 70°. Samples were prepared by pressing the powder onto a slide. Dynamic light scattering (DLS) measurements and zeta potentials of CaF_2_:Y,Gd,Nd NPs dispersed in water were measured using a Malvern ZetaSizer 2000 (Malvern, UK) at room temperature (~25 °C). Fourier transform infrared (FT-IR) spectra were recorded using IRSpirit FTIR spectrophotometers (Shimadzu, Kyoto, Japan). Powder samples (without potassium bromide) were averaged over 15 scans at room temperature with a resolution of 4 cm^−1^. The diameter and morphology of the synthesized CaF_2_:Y,Gd,Nd NPs were characterized by Tecnai 12 Twin (FEI Company, Hillsboro, OR, USA) transmission electron microscopy (TEM) using a OneView camera Model 1095 (Gatan, Pleasanton, CA, USA) at an accelerating voltage of 120 kV. Samples were prepared by adding 1 mg/mL of CaF_2_:Y,Gd,Nd aqueous solution to the surface of glow-discharged copper grids. For scanning electron microscopy (SEM) inspection, CaF_2_:Y,Gd,Nd NPs were mounted on specimen stubs by means of carbon tape. Next, stubs with sample were mounted in an Apreo S LoVac SEM (Thermo Scientific, USA) equipped with an UltraDry energy-dispersive X-ray spectroscopy (EDS) detector (Thermo Scientific, USA). EDS of NPs was performed at 1500× magnification with 30 kV and 51 nA. Emission spectra of NPs at different powers were measured using a Fluorolog^®^-3 with FluorEssence™ spectrometer (Horiba, Kyoto, Japan), and the samples were placed in a quartz cell. UV and Visible spectra were recorded by an Agilent 8453 UV-visible Spectrometer (absorption, Agilent, Santa Clara, CA, USA) and a SpectraMax^®^ iD3 Multi-Mode Microplate Reader (Molecular Devices, San Jose, CA, USA) and aqueous NP solutions (10 mg/mL) were measured in 96-well plates. Vibrating-sample magnetometry (VSM) of the CaF_2_:Y,Gd,Nd NPs was measured on a Quantum Design Versalab physical property measurement system with the VSM option (Quantum Design, San Diego, CA, USA). Data analysis and processing was performed using Zetasizer Software (Version 7.13), GraphPad Prism 8 (GraphPad Software, San Diego, CA, USA), and Origin 8.5 (OriginLab Corporation, Northampton, MA, USA).

### 2.4. Stability of CaF_2_:Y,Gd,Nd NPs

A total of 2 mg CaF_2_:Y,Gd,Nd NPs was dissolved in 10 mL of 50% FCS solution. The samples were mixed thoroughly and placed in a shaker at 37 °C. The particle size and zeta potential of CaF_2_:Y,Gd,Nd NPs in the solvent were measured at different time points (0 h, 1 d, 2 d, 3 d, 4 d, 5 d, 6 d, 7 d) using a Malvern ZetaSizer 2000 (Malvern, UK). Data were processed using Zetasizer Software (Version 7.13, Malvern, UK).

### 2.5. Hemolysis of CaF_2_:Y,Gd,Nd NPs

To investigate the hemolytic potential of CaF_2_:Y,Gd,Nd NPs, an in vitro hemolysis test was carried out. Briefly, a total of 100 μL of fresh blood was collected from the tail vein of BALB/c mice using a vacuum blood collection tube. Then, collected blood was centrifuged at 0.9× *g* for 15 min to remove the serum and then washed 4 times with PBS to obtain hemoglobin. The hemoglobin was diluted in 7 portions with Ca^2+^/Mg^2+^-free PBS and aliquoted in 1.5 mL centrifuge tubes. The supernatant was removed by centrifugation at 0.9× *g* for 5 min. Two groups were randomly removed as negative and positive controls. The group resuspended in 500 μL saline was used as the negative control and the group resuspended in 500 μL 1% Triton X-100 (*v*/*v*) was used as the positive control. Blood cells in the experimental groups were resuspended in isotonic saline containing different concentrations (1, 0.5, 0.25, 0.125, 0.0625 mg/mL) of CaF_2_:Y,Gd,Nd NPs. Samples were gently shaken in a shaker incubator at 37 °C for 4 h. Samples were then centrifuged at 0.9× *g* and 4 °C for 15 min and the supernatant was transferred to a 96-well plate. The absorbance was measured at 540 nm using an enzyme marker (Molecular Devices SpectraMax, San Jose, CA, USA). The hemolysis values of the samples at 540 nm were calculated as follows:

Hemolysis ratio % = (OD sample − OD negative)/(OD positive − OD negative) × 100

### 2.6. MTS Cytotoxicity Assay of CaF_2_:Y,Gd,Nd NPs

In vitro cytotoxicity of CaF_2_:Y,Gd,Nd NPs was studied on 4T1 cells. The 4T1 cells (1 × 10^4^) were inoculated in 96-well plates, 100 μL of medium was added to each well, and the cells were allowed to adhere overnight. The medium was then replaced with DMEM containing different concentrations of CaF_2_:Y,Gd,Nd NPs (2000, 1000, 500, 250, 125, 62.5, and 31.25 µg/mL). The medium was removed at the indicated intervals (24, 48, and 72 h) and cell viability was assessed by MTS (Promega, Madison, WI, USA) according to the manufacturer’s instructions. The absorbance (OD) was measured at 490 nm using an enzyme marker (Molecular Devices, San Jose, CA, USA). Data are expressed as a percentage of the experimental group versus the control group. The experiment was performed in quadruplicate.

### 2.7. Uptake of CaF_2_:Y,Gd,Nd NPs

Cellular uptake of CaF_2_:Y,Gd,Nd NPs was examined by a SP8 LIGHTNING Confocal Microscope (Leica Microsystems, Wetzlar, Germany) and flow cytometry (Becton Dickinson, San Jose, CA, USA). Confocal microscopy was used to demonstrate the uptake and distribution of CaF_2_:Y,Gd,Nd NPs within 4T1 cells. The 4T1 cells were inoculated in 24-well plates containing coverslips. When the cells reached 50–60% confluence, they were incubated with 1 mL of medium containing 250 µg/mL CaF_2_:Y,Gd,Nd NPs for 1, 4, 24, and 48 h. After incubation, the cells were washed 3 times with cold PBS, fixed with 200 μL of 4% paraformaldehyde at room temperature for 20 min, washed twice with cold PBS, treated with 0.1% Triton-X in PBS at room temperature for 15 min, then stained with ghost cyclopeptides for 40 min, and nuclei were stained with DAPI for 5 min. After staining, the cells were viewed and imaged using a SP8 LIGHTNING Confocal Microscope. For flow cytometry, 3 × 10^4^ 4T1 cells/well were inoculated in 24-well plates for 24 h. After incubation, the medium was replaced with 1 mL of medium containing 250 µg/mL CaF_2_:Y,Gd,Nd NPs and incubated at 37 °C for 1, 4, 24, and 48 h. The cells were then washed 5 times with PBS to remove excess NPs, trypsin digested, resuspended with 100 μL FACS buffer (PBS/0.5% BSA/0.02% sodium azide), and the NP fluorescence was analyzed by LSRFortessa FACS Analyzer (BD Biosciences, Franklin Lakes, NJ, USA) to assess the uptake of NPs by the 4T1 cells.

### 2.8. In Vitro Dendritic Cell (DC) Activation Study

To assess the immunogenicity of CaF_2_:Y,Gd,Nd NPs, D1 DCs were inoculated into 96-well plates and incubated with different concentrations (0–125 µg/mL) of CaF_2_:Y,Gd,Nd NPs in an incubator at 37 °C for 24 h. Supernatants were collected for IL-12 assay using an IL-12p40 sandwich ELISA kit (Biolegend, San Diego, CA, USA) and the remaining cells were assessed by flow cytometry to determine the expression of co-stimulatory markers CD86, CD40, and MHC-II. Briefly, D1 DCs were harvested with PBS/EDTA, washed with FACS buffer, and stained with anti-CD40-APC, anti-CD86-FITC, and anti-MHC II-PE. After 30 min of incubation, the cells were washed again with FACS buffer to remove excess antibodies and the stained cells were resuspended in 100 µL FACS buffer. CD86, CD40, and MHC-II expression were measured with an LSR-II cytometer (BD Biosciences, Franklin Lakes, NJ, USA) and analyzed using FlowJo software (version 10).

### 2.9. In Vitro and Ex Vivo Imaging of CaF_2_:Y,Gd,Nd NPs

This study was performed in line with the principles of the Dutch Animal Ethical Commission under license number AVD116008045 and approved by the Animal Experimental Committee from the Leiden University Medical Center (LUMC).

#### 2.9.1. NIR-II Imaging

To evaluate the NIR-II luminescence properties of CaF_2_:Y,Gd,Nd NPs, we used the KIS NIR-II optical imaging system (Kaer Labs, Nantes, France). The InGaAs camera was cooled to −20 °C with a mid-gain setting. The CaF_2_:Y,Gd,Nd NPs solution (2 mg/mL) was excited using an 808 nm laser with a laser power of 50 mW/cm^2^ to obtain images at different wavelengths. The images were recorded by the KIS NIR-II system.

#### 2.9.2. Photoacoustic Imaging

A Vevo 3100 LAZR-X (FUJIFILM VisualSonics, Toronto, ON, Canada) equipped with an MX550D transducer was used to acquire in vitro and ex vivo PA images of CaF_2_:Y,Gd,Nd NPs solutions (10 mg/mL) at 808 nm. In vitro, we measured the intensity of the PAI signal of CaF_2_:Y,Gd,Nd NP solution (10 mg/mL), with water as a control, and recorded PA images. Ex vivo, we first took PA images of BALB/c mouse cadavers before injection, and then injected the CaF_2_:Y,Gd,Nd NP solution (10 mg/mL) into the abdomen of mice and collected PA images immediately after injection. The measurement procedure was performed with a center transmission of 40 MHz and an axial resolution of 40 μm. Data were analyzed using Vevo LAB 5.5.0 (FUJIFILM VisualSonics, Toronto, ON, Canada).

#### 2.9.3. MRI Studies

To determine that the CaF_2_:Y,Gd,Nd NPs were magnetic, MRI measurements were performed on a 7T Bruker BioSpec (Ettlingen, Germany) with a 38 mm transmit/receive birdcage coil. CaF_2_:Y,Gd,Nd gels were configured using 5% agarose solution at different concentrations (0 mg/mL, 1 mg/mL, 2.5 mg/mL, 5 mg/mL, 7.5 mg/mL, 10 mg/mL). Then, the samples were placed in a circular test tube for testing, and the T1-weighted images were obtained using the following parameters: the repetition time/echo time (TR/TE) was 500/11 ms, the number of excitations (NEX) was 4, the field of view (FoV) was 40 × 40 mm^2^, and the Flipangle (FA) was 180. Next, to test the MRI properties of NPs in a biological environment, we injected 100 µL of CaF_2_:Y,Gd,Nd NPs (10 mg/mL) into C57BL/6J mouse cadavers via subcutaneous injection. Images were obtained before and after injection using a small animal coil. The relevant parameters were as following: TR/TE = 10/2.8 ms, FoV = 40 × 40 mm^2^, matrix = 256 × 256. The attenuation images and results were analyzed with ParaVision 360 (Version 2.0. pl.1, Bruker, Germany) software.

### 2.10. Statistical Analysis

Statistical analysis was performed using GraphPad Prism software 8 (GraphPad Software, San Diego, CA, USA). Statistical significance of differences is indicated as * *p* < 0.05, ** *p* < 0.01, *** *p* < 0.001 and **** *p* < 0.0001.

## 3. Results and Discussion

Firstly, we synthesized small-sized CaF_2_:Y,Gd,Nd NPs in a sealed environment under high temperature and pressure using the hydrothermal method developed by Pedroni et al. [54]. The unique physicochemical properties of obtained NPs confer superior optical, magnetic, and photoacoustic characteristics. To better understand the relevant properties of this multifunctional material, we investigated the morphology, biocompatibility, and multimodal imaging aspects of CaF_2_:Y,Gd,Nd NPs in detail.

**Figure 2:**
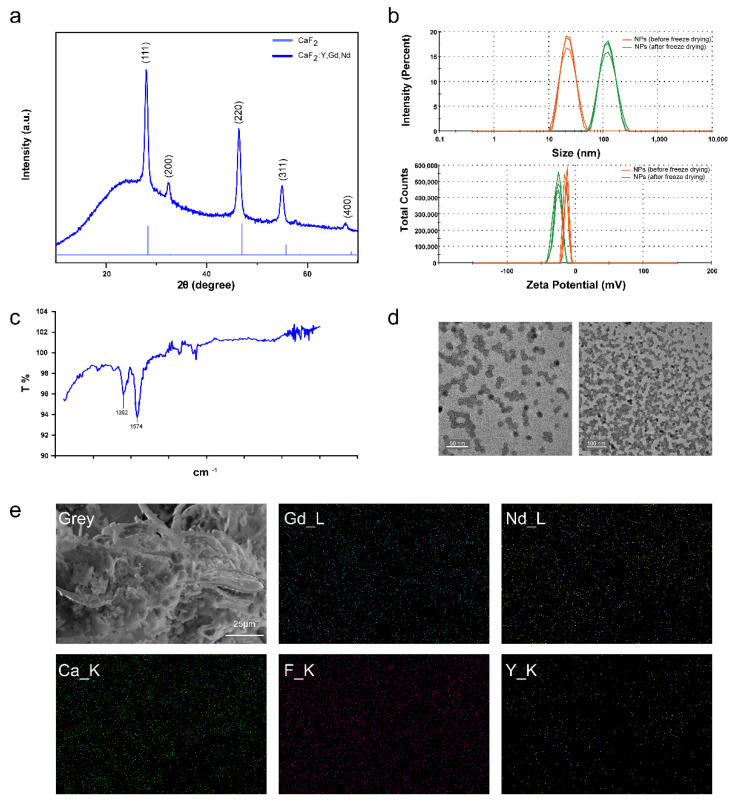
Morphology of CaF_2_:Y,Gd,Nd NPs. (**a**) XRD patterns of CaF_2_:Y,Gd,Nd NPs, and CaF_2_; (**b**) DLS image of CaF_2_:Y,Gd,Nd NPs (1 mg/mL) before and after freeze drying. Measurements were repeated three times; (**c**) FTIR image of CaF_2_:Y,Gd,Nd NPs; (**d**) TEM images of water-soluble CaF_2_:Y,Gd,Nd NPs; (**e**) SEM-EDS elemental mapping images of CaF_2_:Y,Gd,Nd NPs. Scale bar = 25 µm.

To investigate the morphology of CaF_2_:Y,Gd,Nd NPs, we first tested the structure of CaF_2_:Y,Gd,Nd NPs using XRD, as shown in Figure 2a. In NP mapping, the diffraction peaks out of (111), (220), (311), and (400) correspond well to the CaF_2_ standard card (JCPDS 35-0816) in the cubic phase. Previous studies have shown that the appearance of the (200) diffraction peak is the primary indication for doping of the CaF_2_ crystals with rare-earth ions [52,55]. Therefore, we can determine that the main structure of the NPs is a fluorite-type structure (space group *Fm3m*) of CaF_2_ [56,57]. On the other hand, the absence of other spurious peaks also indicates that the CaF_2_:Y,Gd,Nd NPs are in a pure cubic phase. By analyzing the XRD patterns, the crystallite size of CaF_2_:Y,Gd,Nd NPs was calculated as 11.76 ± 1.31 nm using Sherrer formula [58,59]. As this size determination might be susceptible to the influence of the instrument and the sample (microcrystal size, microstrain, etc.), we next determined the diameter of the NPs experimentally.

We found that CaF_2_:Y,Gd,Nd NPs can be easily dispersed in water to form a stable and transparent solution. To assess the effect of NPs in the colloidal phase and their aggregates, we determined their size and zeta potential. The results showed that after freeze drying, CaF_2_:Y,Gd,Nd NPs displayed a significant size increase from 24.17 ± 0.3005 nm to 113.4 ± 43.13 nm, with a small reduction in the zeta potential (−12.5 ± 1.15 mV before to −25.7 ± 0.462 mV after freeze drying) (Figure 2b). The increase in NP size might be caused by aggregation of the NPs due to the freeze-drying process. The high negative zeta potential values, likely caused by the negative groups on the NP surface, indicate a stable system of NPs. Obtaining stable colloidal systems is particularly important in nanomedicine applications [60]. To confirm this speculation, we performed FTIR tests on the sample powder and the appearance of absorption peaks at 1392 cm^−1^ and 1574 cm^−1^ indicated that CaF_2_:Y,Gd,Nd NPs have carboxyl groups on the surface, thereby confirming our speculation (Figure 2c). To further investigate the morphology, we visualized our CaF_2_:Y,Gd,Nd NPs by means of TEM. The TEM images showed a more regular paving-stone-like shape with a particle diameter of 12.13 ± 1.72 nm and an excellent crystallinity which is consistent with the XRD results (Figure 2a,d). Furthermore, EDS was applied for the elemental analysis of freeze-dried CaF_2_:Y,Gd,Nd NPs, as shown in Supplementary Materials Figure S1. The EDS spectrum showed the peaks of Ca, F, Y, Gd, Nd, and the absence of other impurities. In addition, SEM-EDS elemental mapping revealed that these elements were evenly distributed within the NPs (Figure 2e). These results prove that Ca, F, Y, Gd, Nd elements, and rare earth ions (Y^3+^, Gd^3+^, Nd^3+^) were successfully doped into the CaF_2_ matrix. Taken together, our results confirm the successful synthesis of CaF_2_:Y,Gd,Nd NPs.

**Figure 3:**
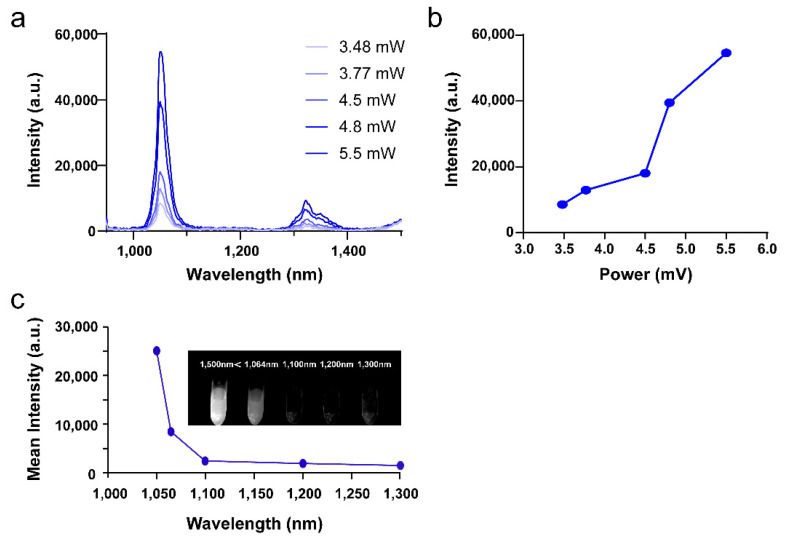
Optical properties of CaF_2_:Y,Gd,Nd NPs. (**a**) The emission spectra of CaF_2_:Y,Gd,Nd NPs with different laser powers (excited by an 808 nm laser); (**b**) the emission intensities of CaF_2_:Y,Gd,Nd NPs (at 1050 nm) with different laser powers under 808 nm laser excitation; (**c**) in vitro NIR-II emission intensity of CaF_2_:Y,Gd,Nd NPs (2 mg/mL) at different wavelengths (excited by an 808 nm laser, 50 mW/cm^2^); inset shows the NIR-II image of CaF_2_:Y,Gd,Nd NPs versus 808 nm excitation.

The key point to determine whether a material can be used as a NIR-II probe is the NIR-II optical property of the material. Based on the results of previous studies [61,62], we tested the emission spectra of CaF_2_:Y,Gd,Nd NPs in the range of 900–1500 nm using an 808 nm laser as the excitation light source, as shown in Figure 3a. The main emission peaks of our NPs were at 1050 nm and 1330 nm, which correspond to the electron transition of Nd^3+^ from the excited state ^4^F_3/2_ energy level to ^4^I_11/2_, and ^4^I_13/2_ levels, respectively [63,64]. Meanwhile, the increase in laser power significantly increased the NIR-II luminescence intensity of Nd^3+^ (Figure 3b). To investigate the NIR-II imaging performance of our NPs, we tested the imaging performance of 2 mg/mL NP solution at different wavelengths. By analyzing the obtained NIR-II image data, we found that CaF_2_:Y,Gd,Nd NPs exhibit a high contrast at 1050 nm (Figure 3c). This result is consistent with the emission spectra we obtained. Thus, we can determine that our NPs can be used as NIR-II imaging probes.

In addition, to better assess the optical properties in the UV and visible range, we tested the absorption of the NPs using an Agilent 8453 UV-visible Spectrometer and a SpectraMax^®^ iD3 Multi-Mode Microplate Reader. As shown in Figure S2a, absorption at the wavelengths 640 nm and 660 nm was detected. After excitation with 620 nm, the NPs showed significant emission at the range of 680–850 nm, thus allowing imaging of the NPs by means of confocal microscopy (Figure S2b).

**Figure 4:**
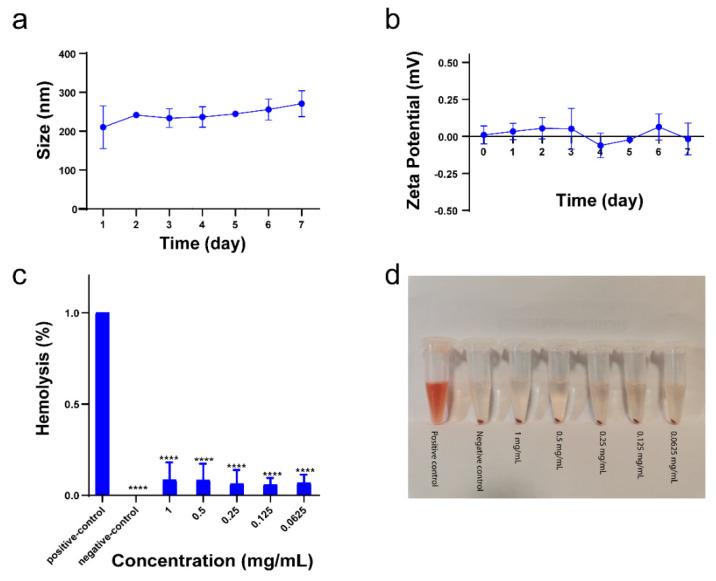
Stability and hemolysis of CaF_2_:Y,Gd,Nd NPs. (**a**) Size and (**b**) zeta potential of CaF_2_:Y,Gd,Nd NPs in 50% FCS; (**c**) hemolysis of CaF_2_:Y,Gd,Nd NPs after incubation with red blood cells at various concentrations (0−1 mg/mL) for 4 h; (**d**) hemolysis image after centrifugation. All experiments were conducted in three independent experiments and data are expressed as mean ± SD. Statistical significance was calculated by comparing the experimental group with the control group using one-way ANOVA (**** *p* < 0.0001).

The stability and hemolysis properties of NPs are important factors in determining whether NPs can be used for bio-diagnostics. When NPs enter the blood stream, the interaction of the NPs with blood components may affect the aggregation of NPs at the target site. Therefore, the stability of an ideal NP-based imaging probe is not disturbed in a complex biological environment and the imaging probe can be delivered to the target site with maximal efficacy, rather than being toxic to blood components. To investigate the stability of our NPs under physiological conditions, the NPs were resuspended in medium containing 50% FCS, and their size and charge was characterized by DLS over a period of 7 days. We found that the size of all CaF_2_:Y,Gd,Nd NPs increased compared to NPs dissolved in water (Figure 2b and Figure 4a), and the zeta potential of CaF_2_:Y,Gd,Nd NPs shifted to a higher positive charge with a potential close to 0 mV (Figure 4b). This phenomenon may result from the adsorption of cations or serum proteins in solution by the negative charge on the surface of the NPs [65,66]. To assess the toxicity of NPs on blood cells, we performed hemolysis experiments. After incubation of erythrocytes with NPs at different concentrations for 4 h, as shown in Figure 4c,d, the percentage of hemolysis was close to 0 for all samples compared to the non-hemolyzed positive control group. This result effectively proves that CaF_2_:Y,Gd,Nd NPs do not cause toxicity to erythrocytes. In conclusion, our CaF_2_:Y,Gd,Nd NPs can be used for bio-diagnostics.

**Figure 5:**
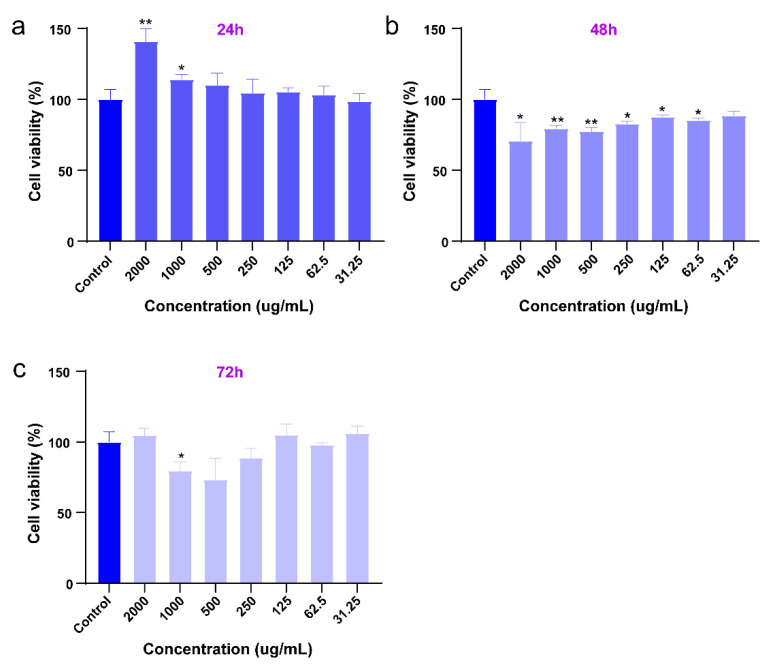
Viability of 4T1 cells incubated with CaF_2_:Y,Gd,Nd NPs at increasing concentrations (0−2 mg/mL) after (**a**) 24; (**b**) 48; and (**c**) 72 h assessed by MTS assay. All data are expressed as mean ± SD from three independent experiments. Statistical significance was calculated using the *t*-test, by comparing experimental groups to the control group (* *p* < 0.05, ** *p* < 0.01).

Assessing the toxicity of CaF_2_:Y,Gd,Nd NPs is an important indicator to ensure their biosafety. Here, 4T1 cells were used as an assessment model and 4T1 cells were incubated with different concentrations of NPs for 24, 48, and 72 h and then analyzed by MTS assay. In Figure 5, in comparison with the control group, we found that CaF_2_:Y,Gd,Nd NPs barely affected the metabolic activity of 4T1 cells 24 h after incubation, while the metabolic activity was decreased to 75% at concentrations of 500 µg/mL and 1000 µg/mL after 48 h. After 72 h of incubation, the metabolic activity of 4T1 cells decreased only at a NP concentration of 1000 µg/mL, while lower concentrations did not induce any cytotoxic effects. While our results show that CaF_2_:Y,Gd,Nd NPs did not exhibit cytotoxicity, further in vivo toxicity studies are needed to determine whether CaF_2_:Y,Gd,Nd NPs are suitable for applications in living organisms.

**Figure 6:**
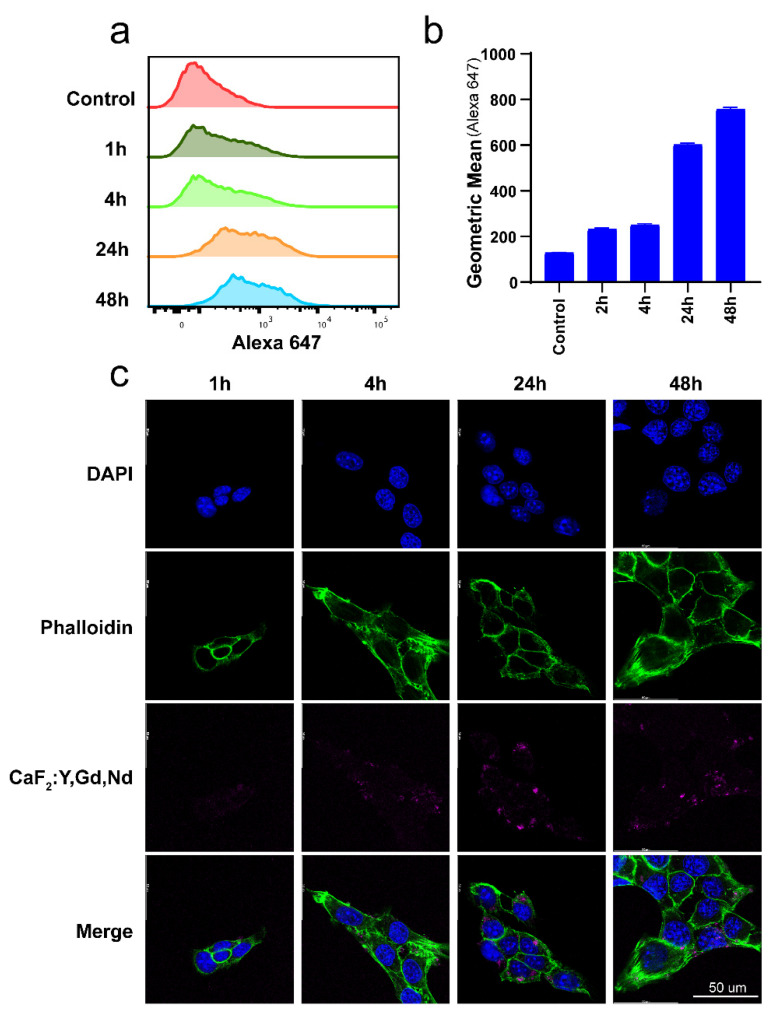
Uptake of CaF_2_:Y,Gd,Nd NPs by 4T1 cells. (**a**) Representative flow cytometric plots and (**b**) flow cytometric analysis of CaF_2_:Y,Gd,Nd NPs uptake by 4T1 cells; (**c**) confocal microscope images of CaF_2_:Y,Gd,Nd NPs uptake after 1, 4, 24, and 48 h. Cell nuclei are stained blue (DAPI), actin cytoskeleton is green (Phalloidin), and CaF_2_:Y,Gd,Nd NPs are displayed in magenta. Scale bar = 50 µm.

CaF_2_:Y,Gd,Nd NPs’ optical characteristics partially overlap with the excitation and emission spectra of dyes used in flow cytometry and confocal microscopy, such as Alexa Fluor 647. Thus, we explored whether CaF_2_:Y,Gd,Nd NPs could be used directly to measure the cellular uptake of NPs by flow cytometry and confocal microscopy (Figure 2d,e). Rare-earth NPs represent a promising tool for the early detection and treatment of cancer. For breast cancer in particular, the development of new imaging probes is desperately needed, because mammography, the standard diagnosis method, often leads to false-negative results and therefore to therapeutic delays [67]. In order to ensure that CaF_2_:Y,Gd,Nd NPs could be taken up efficiently by breast cancer cells to achieve high-contrast bioimaging, we first analyzed the uptake of CaF_2_:Y,Gd,Nd NPs by 4T1 cells at different time points by using flow cytometry. As shown in Figure 6a, compared to the control group, the uptake efficiency of NPs was low during a short period of time, but gradually increased with time. To verify this conclusion, we used confocal microscopy to visualize the uptake of CaF_2_:Y,Gd,Nd NPs. As shown in Figure 6b, the signal of CaF_2_:Y,Gd,Nd NPs (magenta) was barely observed after 1 h of incubation, while a larger amount of signal could be observed as the incubation time increased. This result was consistent with the results obtained by flow cytometry. Therefore, we can conclude that CaF_2_:Y,Gd,Nd NPs were effectively taken up by 4T1 cells. Moreover, CaF_2_:Y,Gd,Nd NPs were intrinsically monitorable by flow cytometry and confocal imaging.

**Figure 7:**
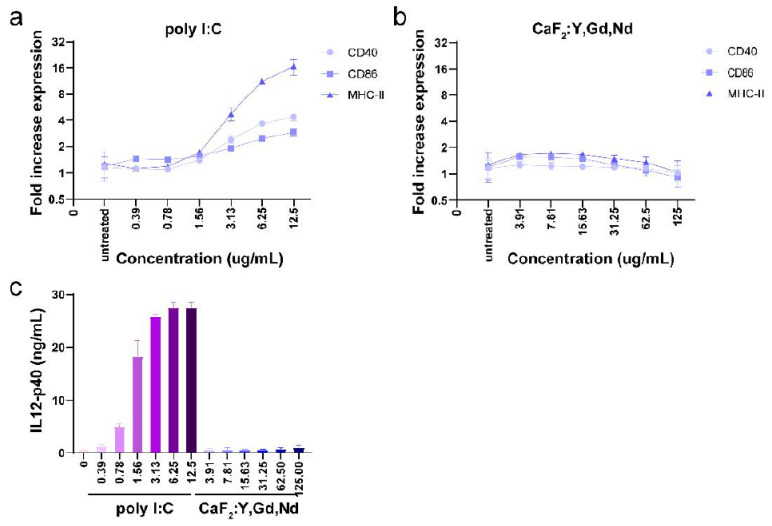
Immunogenicity of CaF_2_:Y,Gd,Nd NPs measured by flow cytometry and IL-12 ELISA. D1 DCs were incubated with different concentrations of CaF_2_:Y,Gd,Nd NPs for 24 h. (**a**) Expression of CD40, CD86, and MHC-II in poly I:C group (compared to untreated group); (**b**) expression of CD40, CD86, and MHC-II in CaF_2_:Y,Gd,Nd NPs group (compared to untreated group); (**c**) production of IL-12 (ng/mL) was measured using ELISA for poly I:C group and CaF_2_:Y,Gd,Nd NPs group. Data are expressed as mean ± SD for three independent experiments.

Understanding whether NPs will interact with the immune system is as important as analyzing their hemolytic properties. It is well known that immune cells in blood and tissues have a tendency to phagocytose and eliminate certain NPs, which may lead to artificial activation of the immune system and cause undesirable systemic side effects. Thus, a good imaging probe must be “invisible” to immune cells. This attribute allows nanoparticles to remain in circulation for a prolonged time without being cleared by immune cells [68]. To assess whether CaF_2_:Y,Gd,Nd NPs induce an immune response in the body, we incubated D1DCs with NPs at different concentrations (0–125 µg/mL). After 24 h, we measured the expression of co-stimulatory markers CD86, CD40, and MHC-II and the production of the pro-inflammatory cytokine IL-12. As shown in Figure 7b, the presence of CaF_2_:Y,Gd,Nd NPs did not induce the expression of CD86, CD40, and MHC-II compared to the positive control treated with TLR-3 ligand poly (I:C) (Figure 7a). Additionally, the supernatant was collected and IL-12 production was assessed using ELISA. We found that NPs did not induce the production of the pro-inflammatory cytokine IL-12 at any concentration (Figure 7c). Overall, these studies indicate that CaF_2_:Y,Gd,Nd NPs are inert and non-immunogenic and can be used as potential bioimaging probes.

**Figure 8:**
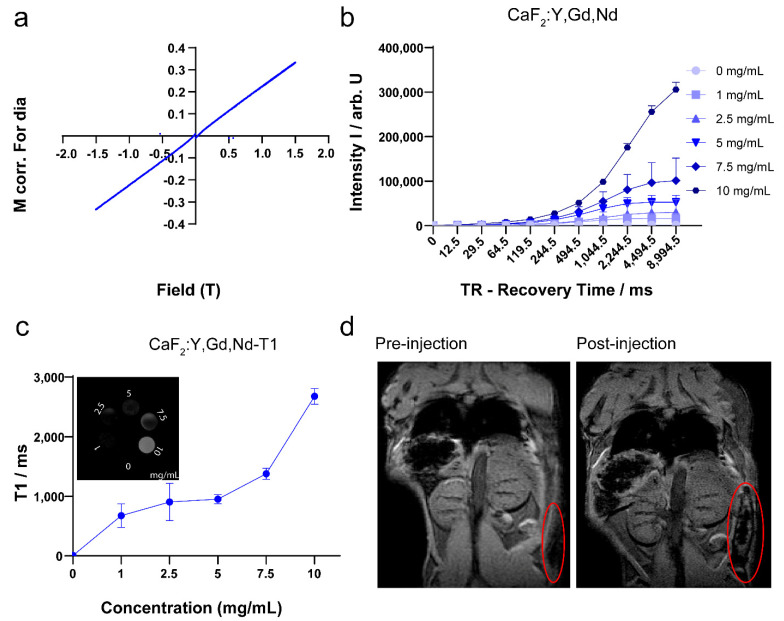

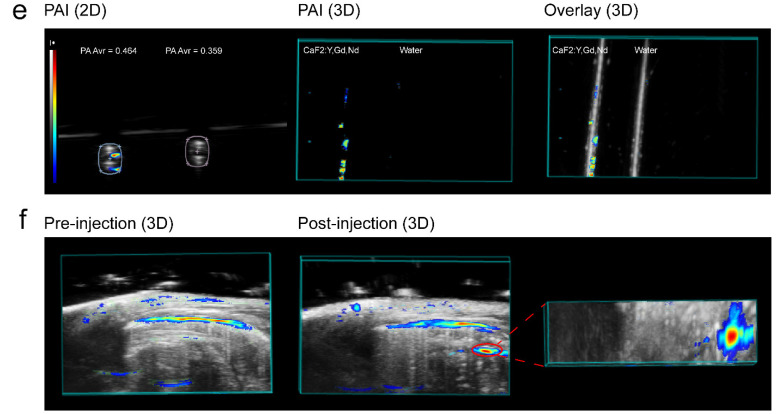
MRI and PAI performance of CaF_2_:Y,Gd,Nd NPs. (**a**) The VSM measurement of CaF_2_:Y,Gd,Nd NPs; (**b**) MRI signal intensity of different concentrations of CaF_2_:Y,Gd,Nd NPs with increasing recovery time; (**c**) in vitro T1 relaxation rates of different CaF_2_:Y,Gd,Nd NP concentrations; inset shows T1-weighted MRI images of CaF_2_:Y,Gd,Nd NPs at different concentrations in 0.5 % agarose gel; (**d**) ex vivo MRI images before and after subcutaneous injection of CaF_2_:Y,Gd,Nd NPs (10 mg/mL) into a mouse cadaver; (**e**) in vitro PA images (2D and 3D) of CaF_2_:Y,Gd,Nd NPs (10 mg/mL), water is the control; (**f**) ex vivo PA images (3D) before and after injection of CaF_2_:Y,Gd,Nd NPs (10 mg/mL) into a mouse. Unspecific signal is caused by endogenous tissue chromophores.

The presence of the element Gd^3+^, which possesses a high atomic number, makes it possible for CaF_2_:Y,Gd,Nd NPs to be used as a contrast agent in MRI [69,70,71]. To verify the magnetic properties of CaF_2_:Y,Gd,Nd NPs, VSM measurement is required. As we expected, the NPs exhibited typical paramagnetic behavior at room temperature (300 K) in an applied magnetic field of 1.5 T, indicating that the NPs are paramagnetically responsive to external magnetic fields (Figure 8a). This is consistent with previous studies, offering the possibility of CaF_2_:Y,Gd,Nd NPs as MR probes [72,73]. Then, T1-weighted MRI scans were performed on agarose-gel-embedded samples with different concentrations of CaF_2_:Y,Gd,Nd NPs. As the results in Figure 8b,c show, the signal intensity of CaF_2_:Y,Gd,Nd NPs was positively correlated with the recovery time, and the analysis shows that the T1 value of the samples was higher and the signal intensity of the T1-weighted MRI increased with increasing sample concentration. Subsequently, to test the MRI performance of CaF_2_:Y,Gd,Nd NPs within a complex environment, CaF_2_:Y,Gd,Nd NPs (10 mg/mL) were injected subcutaneously into a mouse cadaver and a significant signal could be observed at the site of injection by means of MRI (Figure 8d). Therefore, when used as a contrast agent, paramagnetic CaF_2_:Y,Gd,Nd NPs can effectively improve MRI efficiency and sensitivity. Currently, lanthanide-doped NPs are gradually applied to high-contrast PA imaging [74,75]. To investigate this property, we first performed in vitro PA measurements on CaF_2_:Y,Gd,Nd NPs and found that the presence of CaF_2_:Y,Gd,Nd NPs significantly enhanced the PA signal compared to water, with PA amplitudes up to 0.464 AU (arbitrary unit; Figure 8e). On this basis, we further evaluated the potential of CaF_2_:Y,Gd,Nd NPs as a PAI agent by intraperitoneal injection of CaF_2_:Y,Gd,Nd NPs in mouse cadavers. When compared to the PAI signal before injection of the NPs, a distinct PAI signal could be observed at 808 nm at the injection site after injection. The PAI signal of the NPs could be well distinguished from the surrounding tissue (Figure 8f). The success of our ex vivo PAI experiments illustrates that CaF_2_:Y,Gd,Nd NPs can be used as excellent PAI contrast agents for tissue imaging and diagnosis. In addition to their application as NIR/PAI contrast agent, our CaF_2_:Y,Gd,Nd NPs can also be used as a MRI contrast agent.

## 4. Conclusions

Small-sized CaF_2_:Y,Gd,Nd NPs synthesized by a facile hydrothermal method can be used as multimodal NIR-II fluorescence/photoacoustic/magnetic resonance imaging probes. The excellent morphology and NIR-II optical properties provide the basis for NIR-II diagnostic applications of our CaF_2_:Y,Gd,Nd NPs. Biotoxicity and stability analyses have shown that our NPs are biocompatible as well as safe in biological settings. In addition, the fact that immune cells were not activated in response to CaF_2_:Y,Gd,Nd NPs provides favorable leverage for their application in vivo. The doping of Gd^3+^ significantly increased the MRI contrast of CaF_2_:Y,Gd,Nd NPs, thereby enabling accurate MRI diagnostic properties. In addition, the apparent PAI signal offers a wide range of applications for CaF_2_:Y,Gd,Nd NPs in PAI-based diagnostics. Thus, our work not only demonstrates a multifunctional NP with excellent three-mode imaging capability, but also provides a potential solution to the medical system dilemma. A hot trend in current research is to equip rare-earth NPs with therapeutic functions through surface modifications (antibodies, chemotherapeutic agents, or photosensitizers, etc.), while achieving multimodal imaging of tumor sites [76,77,78,79]. In particular, surface modification with tumor-targeting ligands can enhance the potential of rare-earth NPs for cancer diagnosis and treatment [19]. We strongly believe that this study provides an important insight into the design of novel multifunctional nanomaterials as potential therapeutic agents for the treatment and diagnosis of diseases in the future, and helps to drive the clinical translation of novel therapeutic agents.

## Data Availability

Not applicable.

## Supplementary Materials

The following supporting information can be downloaded at: https://www.mdpi.com/article/10.3390/pharmaceutics14040840/s1, Figure S1: EDS spectrum of CaF_2_:Y,Gd,Nd NPs; Figure S2: Absorption and emission spectra of CaF_2_:Y,Gd,Nd NPs in the visible region.
