# Correction: Yu et al. Achieving Effective Multimodal Imaging with Rare-Earth Ion-Doped CaF_2_ Nanoparticles. *Pharmaceutics* 2022, *14*, 840

**DOI:** 10.3390/pharmaceutics16010091

**Published:** 2024-01-09

**Authors:** Zhenfeng Yu, Yuanyuan He, Timo Schomann, Kefan Wu, Yang Hao, Ernst Suidgeest, Hong Zhang, Christina Eich, Luis J. Cruz

**Affiliations:** 1 Translational Nanobiomaterials and Imaging Group, Department of Radiology, Leiden University Medical Center, 2333 ZA Leiden, The Netherlands; z.yu@lumc.nl (Z.Y.); y.he@lumc.nl (Y.H.); t.schomann@lumc.nl (T.S.); y.hao@lumc.nl (Y.H.); 2 Percuros B.V., Zernikedreef 8, 2333 CL Leiden, The Netherlands; 3 Van’t Hoff Institute for Molecular Sciences, University of Amsterdam, Science Park 904, 1098 XH Amsterdam, The Netherlands; k.wu@uva.nl (K.W.); h.zhang@uva.nl (H.Z.); 4 C.J. Gorter Center for High Field MRI, Department of Radiology, Leiden University Medical Center, 2333 ZA Leiden, The Netherlands

## 1. Text Correction

There was an error in the original publication [1]. In the abstract, there is a mistake in the element name.

A correction has been made to “**Abstract**”:

Nowadays, cancer poses a significant hazard to humans. Limitations in early diagnosis techniques not only result in a waste of healthcare resources but can even lead to delays in diagnosis and treatment, consequently reducing cure rates. Therefore, it is crucial to develop an imaging probe that can provide diagnostic information precisely and rapidly. Here, we used a simple hydrothermal method to design a multimodal imaging probe based on the excellent properties of rare-earth ions. Calcium fluoride co-doped with yttrium, gadolinium, and neodymium (CaF_2_:Y,Gd,Nd) nanoparticles (NPs) is highly crystalline, homogeneous in morphology, and displays a high biosafety profile. In addition, in vitro and ex vivo experiments explored the multimodal imaging capability of CaF_2_:Y,Gd,Nd and demonstrated the efficient performance of CaF_2_:Y,Gd,Nd during NIR-II fluorescence/photoacoustic/magnetic resonance imaging. Collectively, our novel diagnosis nanoparticle will generate new ideas for the development of multifunctional nanoplatforms for disease diagnosis and treatment.

There was an error in the original publication [1]. There is a need to correct some details.

A correction has been made to “**2.9.3 MRI Studies**”:

To determine that the CaF_2_:Y,Gd,Nd NPs were magnetic, MRI measurements were performed on a 7T Bruker BioSpec (Ettlingen, Germany) with a 38 mm transmit/receive birdcage coil. CaF_2_:Y,Gd,Nd gels were configured using 1% agarose solution at different concentrations (0 mg/mL, 1 mg/mL, 2 mg/mL, 3 mg/mL, 4 mg/mL, 5 mg/mL). Then, the samples were placed in a circular test tube for testing. T_1_ relaxation was measured using a saturation recovery sequence with the following parameters: 9 repetition times (TR) of 18, 35, 70, 125, 250, 500, 1050, 2250, 4500, 9000 ms, echo time (TE) 6 ms, field of view (FoV) 30 × 30 mm^2^, matrix 64 × 64, slice thickness 2 mm. T_2_ relaxation was measured using a multi spin echo sequence with the following parameters: TR 2200 ms, TE and echo spacing 6.5 ms, 25 echoes, FoV 30 × 30 mm^2^, matrix 64 × 64, slice thickness 2 mm. Next, to test the MRI properties of NPs in a biological environment, we injected 100 µL of CaF_2_:Y,Gd,Nd NPs (10 mg/mL) into C57BL/6J mouse cadavers via subcutaneous injection. Images were obtained before and after injections. The relevant parameters were as follows: gradient echo sequence with TR/TE = 10/2.8 ms, FoV = 40 × 40 mm^2^, matrix = 256 × 256. The attenuation images and results were analyzed with ParaVision 360 (Version 2.0. pl.1, Bruker, Germany) software. Relaxation times were determined using standard mono-exponential functions.

There was an error in the original publication [1]. The figure numbers are incorrectly labelled.

A correction has been made to “**3. Results and Discussion**, Paragraph 8”:

CaF_2_:Y,Gd,Nd NPs’ optical characteristics partially overlap with the excitation and emission spectra of dyes used in flow cytometry and confocal microscopy, such as Alexa Fluor 647. Thus, we explored whether CaF_2_:Y,Gd,Nd NPs could be used directly to measure the cellular uptake of NPs using flow cytometry and confocal microscopy (Figure S2a,b). Rare-earth NPs represent a promising tool for the early detection and treatment of cancer. For breast cancer in particular, the development of new imaging probes is desperately needed, because mammography, the standard diagnosis method, often leads to false-negative results and therefore to therapeutic delays [67]. In order to ensure that CaF_2_:Y,Gd,Nd NPs could be taken up efficiently by breast cancer cells to achieve high-contrast bioimaging, we first analyzed the uptake of CaF_2_:Y,Gd,Nd NPs by 4T1 cells at different time points using flow cytometry. As shown in Figure 6a,b, compared to the control group, the uptake efficiency of NPs was low during a short period of time, but gradually increased with time. To verify this conclusion, we used confocal microscopy to visualize the uptake of CaF_2_:Y,Gd,Nd NPs. As shown in Figure 6c, the signal of CaF_2_:Y,Gd,Nd NPs (magenta) was barely observed after 1 h of incubation, while a larger amount of signal could be observed as the incubation time increased. This result was consistent with the results obtained by flow cytometry. Therefore, we can conclude that CaF_2_:Y,Gd,Nd NPs were effectively taken up by 4T1 cells. Moreover, CaF_2_:Y,Gd,Nd NPs were intrinsically monitorable using flow cytometry and confocal imaging.

There was an error in the original publication [1]. We have discovered errors in the reporting and interpretation of the MRI properties of the nanoparticles.

A correction has been made to “**3. Results and Discussion**, Paragraph 10”:

The presence of the element Gd^3+^, which possesses a high atomic number, makes it possible for CaF_2_:Y,Gd,Nd NPs to be used as a contrast agent in MRI [69–71]. To verify the magnetic properties of CaF_2_:Y,Gd,Nd NPs, VSM measurement is required. As we expected, the NPs exhibited typical paramagnetic behavior at room temperature (300 K) in an applied magnetic field of 1.5 T, indicating that the NPs are paramagnetically responsive to external magnetic fields (Figure 8a). This is consistent with previous studies, offering the possibility of CaF_2_:Y,Gd,Nd NPs as MR probes [72,73]. Then, MRI scans were performed on agarose-gel-embedded samples with different concentrations of CaF_2_:Y,Gd,Nd NPs. As the results in Figure 8b,c show, the signal intensity of CaF_2_:Y,Gd,Nd NPs show a faster decay of the T_2_ relaxation, resulting in lower signal intensities (dark contrast) in the T_2_-weighted MR image with increasing sample concentration. T_1_ contrast was less pronounced; for this concentration range, only subtle changes could be observed in the T_1_-weighted MR image. Both the T_1_ relaxation rate and T_2_ relaxation rate increased linearly with Gd concentration (R_1_^2^ = 0.9388, R_2_^2^ = 0.9677, respectively). The r_1_ and r_2_ relaxivity values are 0.1059 mM Gd^−1^·s^−1^ and 6.275 mM Gd^−1^·s^−1^; the r_2_/r_1_ ratio for CaF_2_:Y,Gd,Nd NPs is ~59. These indicate that CaF_2_:Y,Gd,Nd NPs is superparamagnetic and functions as a potential T_2_ MRI agent [74]. Subsequently, to test the MRI performance of CaF_2_:Y,Gd,Nd NPs within a complex environment, CaF_2_:Y,Gd,Nd NPs (10 mg/mL) were injected subcutaneously into a mouse cadaver and a significant signal could be observed at the site of injection by means of MRI (Figure 8d). Therefore, when used as a contrast agent, paramagnetic CaF_2_:Y,Gd,Nd NPs can effectively improve MRI efficiency and sensitivity. Currently, lanthanide-doped NPs are gradually applied to high-contrast PA imaging [75,76]. To investigate this property, we first performed in vitro PA measurements on CaF_2_:Y,Gd,Nd NPs and found that the presence of CaF_2_:Y,Gd,Nd NPs significantly enhanced the PA signal compared to water, with PA amplitudes up to 0.464 AU (arbitrary unit; Figure 8e). On this basis, we further evaluated the potential of CaF_2_:Y,Gd,Nd NPs as a PAI agent by intraperitoneal injection of CaF_2_:Y,Gd,Nd NPs in mouse cadavers. When compared to the PAI signal before injection of the NPs, a distinct PAI signal could be observed at 808 nm at the injection site after injection. The PAI signal of the NPs could be well distinguished from the surrounding tissue (Figure 8f). The success of our ex vivo PAI experiments illustrates that CaF_2_:Y,Gd,Nd NPs can be used as excellent PAI contrast agents for tissue imaging and diagnosis. In addition to their application as NIR/PAI contrast agent, our CaF_2_:Y,Gd,Nd NPs can also be used as an MRI contrast agent.

There was an error in the original publication. The conclusion of the MRI performance of nanoparticles is not accurate. 

A correction has been made to “**4. Conclusions**”:

Small-sized CaF_2_:Y,Gd,Nd NPs synthesized using a facile hydrothermal method can be used as multimodal NIR-II fluorescence/photoacoustic/magnetic resonance imaging probes. The excellent morphology and NIR-II optical properties provide the basis for NIR-II diagnostic applications of our CaF_2_:Y,Gd,Nd NPs. Biotoxicity and stability analyses have shown that our NPs are biocompatible as well as safe in biological settings. In addition, the fact that immune cells were not activated in response to CaF_2_:Y,Gd,Nd NPs provides favorable leverage for their application in vivo. The doping of Gd^3+^ shows a stronger increase of transverse relaxation rate (1/T_2_) than the longitudinal relaxation rate (1/T_1_) in CaF_2_:Y,Gd,Nd NPs, thereby enabling accurate T_2_-MR diagnostic properties, which are similar to the previous study [77]. In addition, the apparent PAI signal offers a wide range of applications for CaF_2_:Y,Gd,Nd NPs in PAI-based diagnostics. Thus, our work not only demonstrates a multifunctional NP with excellent three-mode imaging capability, but also provides a potential solution to the medical system dilemma. A hot trend in current research is to equip rare-earth NPs with therapeutic functions through surface modifications (antibodies, chemotherapeutic agents, or photosensitizers, etc.), while achieving multimodal imaging of tumor sites [78–81]. In particular, surface modification with tumor-targeting ligands can enhance the potential of rare-earth NPs for cancer diagnosis and treatment [19]. We strongly believe that this study provides an important insight into the design of novel multifunctional nanomaterials as potential therapeutic agents for the treatment and diagnosis of diseases in the future, and helps to drive the clinical translation of novel therapeutic agents.

The authors state that the scientific conclusions are unaffected. This correction was approved by the Academic Editor. The original publication has also been updated.

## 2. Error in Figure

In the original publication [1], there are mistakes in the legend and image for Figure 8. The legends of Figure 8b,c are inaccurate and need to be corrected. And the original MRI data of Figure 8b,c was incomplete and not analyzed correctly. The corrected Figure 8 appears below.

**Figure 8:**
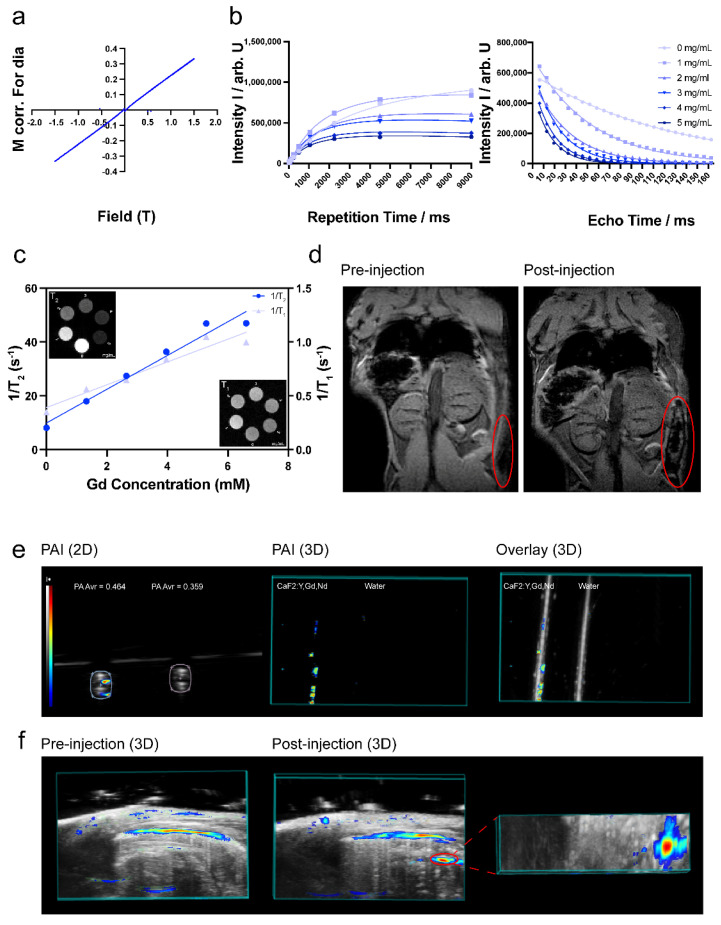
MRI and PAI performance of CaF_2_:Y,Gd,Nd NPs. (**a**) The VSM measurement of CaF_2_:Y,Gd,Nd NPs; (**b**) MRI signal intensity of different concentrations of CaF_2_:Y,Gd,Nd NPs with longitudinal magnetization recovery and transverse magnetization decay; (**c**) in vitro T_1_ and T_2_ relaxation rates of different Gd concentrations; inset shows T_1_-weighted and T_2_-weighted MR images of CaF_2_:Y,Gd,Nd NPs at different concentrations in 1% agarose gel; (**d**) ex vivo MRI images before and after subcutaneous injection of CaF_2_:Y,Gd,Nd NPs (10 mg/mL) into a mouse cadaver; (**e**) in vitro PA images (2D and 3D) of CaF_2_:Y,Gd,Nd NPs (10 mg/mL), water is the control; and (**f**) ex vivo PA images (3D) before and after injection of CaF_2_:Y,Gd,Nd NPs (10 mg/mL) into a mouse. Unspecific signal is caused by endogenous tissue chromophores.

The authors state that the scientific conclusions are unaffected. This correction was approved by the Academic Editor. The original publication has also been updated.

## 3. References

We added new references 74 and 77 as needed. With this correction, the order of some references has been adjusted accordingly. The authors state that the scientific conclusions are unaffected. This correction was approved by the Academic Editor. The original publication has also been updated.

74. Caspani, S.; Magalhães, R.; Araújo, J.P.; Sousa, C.T. Magnetic nanomaterials as contrast agents for MRI. *Materials* **2020**, *13*, 2586.

77. Kadria-Vili, Y.; Neumann, O.; Zhao, Y.; Nordlander, P.; Martinez, G.V.; Bankson, J.A.; Halas, N.J. Gd_2_O_3_-mesoporous silica/gold nanoshells: A potential dual *T*_1_/*T*_2_ contrast agent for MRI-guided localized near-IR photothermal therapy. *Proc. Natl. Acad. Sci.* **2022**, *119*, e2123527119.

The authors state that the scientific conclusions are unaffected. This correction was approved by the Academic Editor. The original publication has also been updated.
