# Supplementary material for: Achieving Effective Multimodal Imaging with Rare-Earth Ion-Doped CaF2 Nanoparticles
Source: Pharmaceutics. 2022 Apr 11;14(4):840. doi: 10.3390/pharmaceutics14040840 (PMC9024546; doi:10.3390/pharmaceutics14040840)
Supplement: Supplementary file 1 [file pharmaceutics-14-00840-s001.zip › pharmaceutics-1649142-supplementary.pdf]

pharmaceutics

Supporting Information:

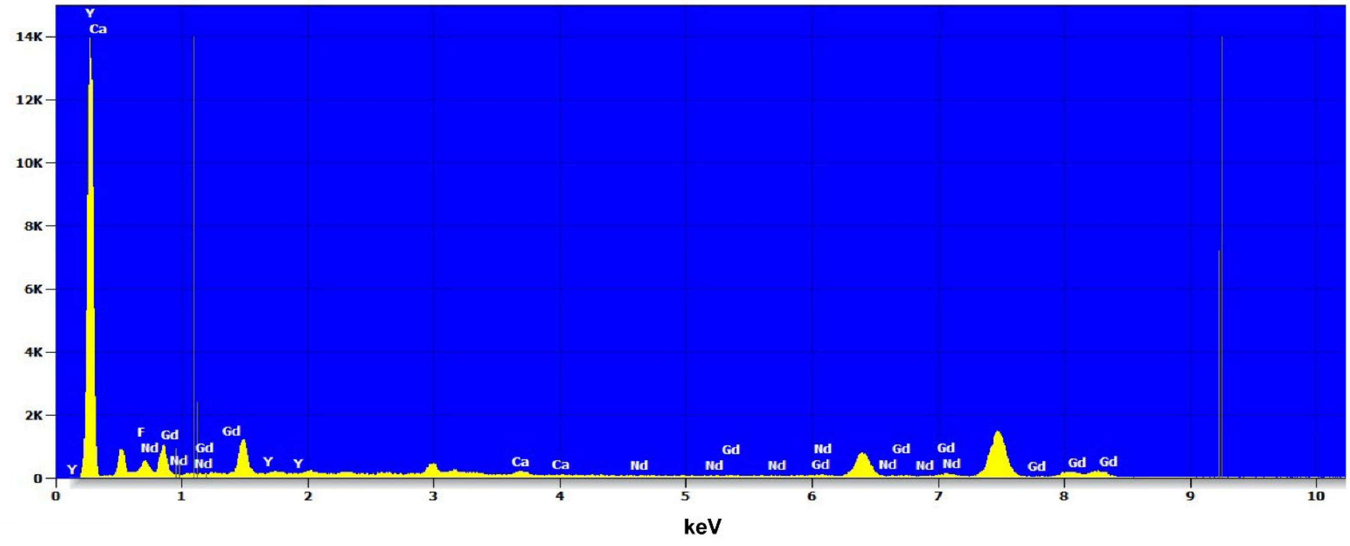

Figure S1. EDS spectrum of CaF_2:Y,Gd,Nd NPs.

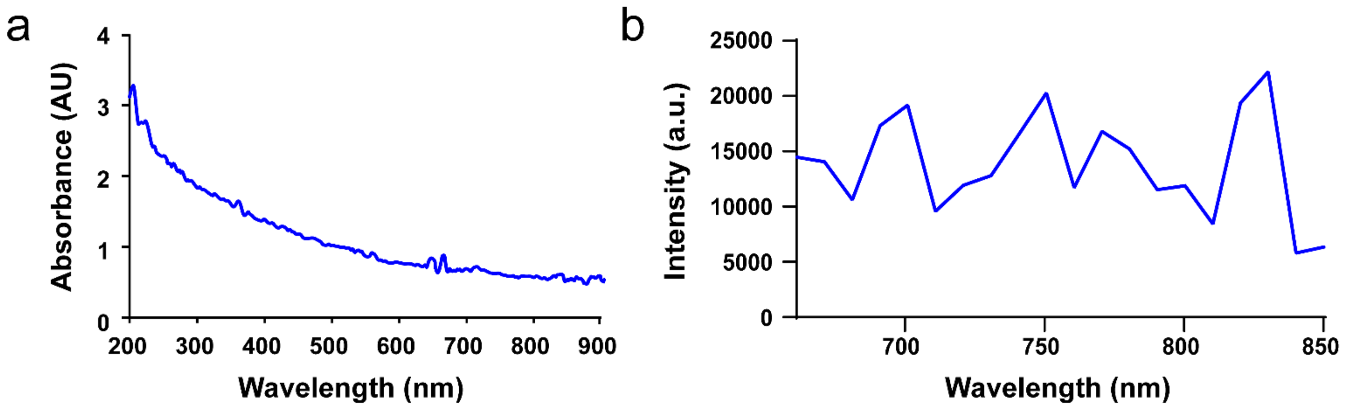

Figure S2. Absorption and emission spectra of CaF_2:Y,Gd,Nd NPs in the visible region. (**a**) The absorption spectra of CaF_2:Y,Gd,Nd solution (10 mg/mL) in the visible region measured with SpectraMax® iD3 Multi-Mode Microplate Reader; (**b**) the emission spectra of CaF_2:Y,Gd,Nd NPs solution (10 mg/mL) in the 680-850 nm region measured with SpectraMax® iD3 Multi-Mode Microplate Reader under 620 nm excitation.
